# Intracranial Hemorrhage Induced Takotsubo Cardiomyopathy: A Case Report

**DOI:** 10.7759/cureus.41718

**Published:** 2023-07-11

**Authors:** Henry Mann, Sindhu C Pokhriyal, Josef Kusayev, Anando Malo, Ajibola M Adedayo

**Affiliations:** 1 Internal Medicine, One Brooklyn Health System Interfaith Medical Center, Brooklyn, USA; 2 Medicine, New York Medical College, Valhalla, USA; 3 Cardiology, One Brooklyn Health System Interfaith Medical Center, Brooklyn, USA

**Keywords:** takotsubo cardiomyopathy, intracranial hemorrhage, hemorrhagic stroke, broken-heart syndrome, stroke, stress induced cardiomyopathy

## Abstract

Takotsubo cardiomyopathy, also known as stress-induced cardiomyopathy or "broken heart syndrome," is a reversible cardiac disorder characterized by left ventricular dysfunction without significant obstructive coronary artery disease. It is classically secondary to emotional stress in postmenopausal women but can also be secondary to physical stress. This report presents a unique case of takotsubo cardiomyopathy induced by intracranial hemorrhage in an 80-year-old female who presented with syncope.

## Introduction

Takotsubo cardiomyopathy (TCM) is defined as acute, reversible left ventricular dysfunction induced by stress, which is classically emotional but can also be physiologic, and most commonly occurs in postmenopausal women [1]. It is diagnosed by the presence of cardiac ischemia with left ventricular dysfunction and the absence of obstructive coronary artery disease. Cardiac ischemia can be demonstrated by ischemic changes on electrocardiogram or elevated troponin, while left ventricular dysfunction or the absence of obstructive coronary artery disease can be demonstrated by coronary angiogram [2]. We present here a rare case of TCM induced by intracranial hemorrhage (ICH) in an 80-year-old female

## Case presentation

**Figure 1:**
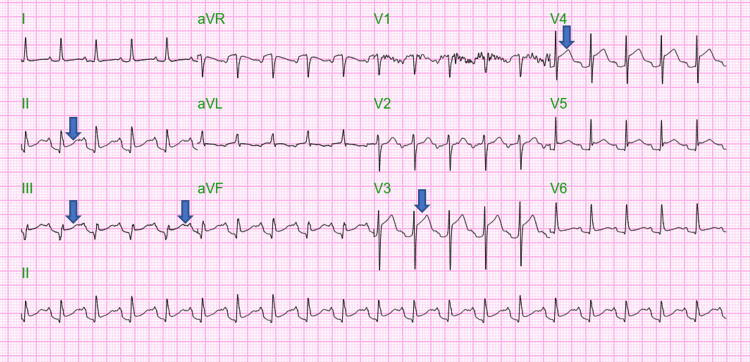
Electrocardiogram on admission revealing ST-segment elevations in leads II, III, aVF, V3, and V4.

**Figure 2:**
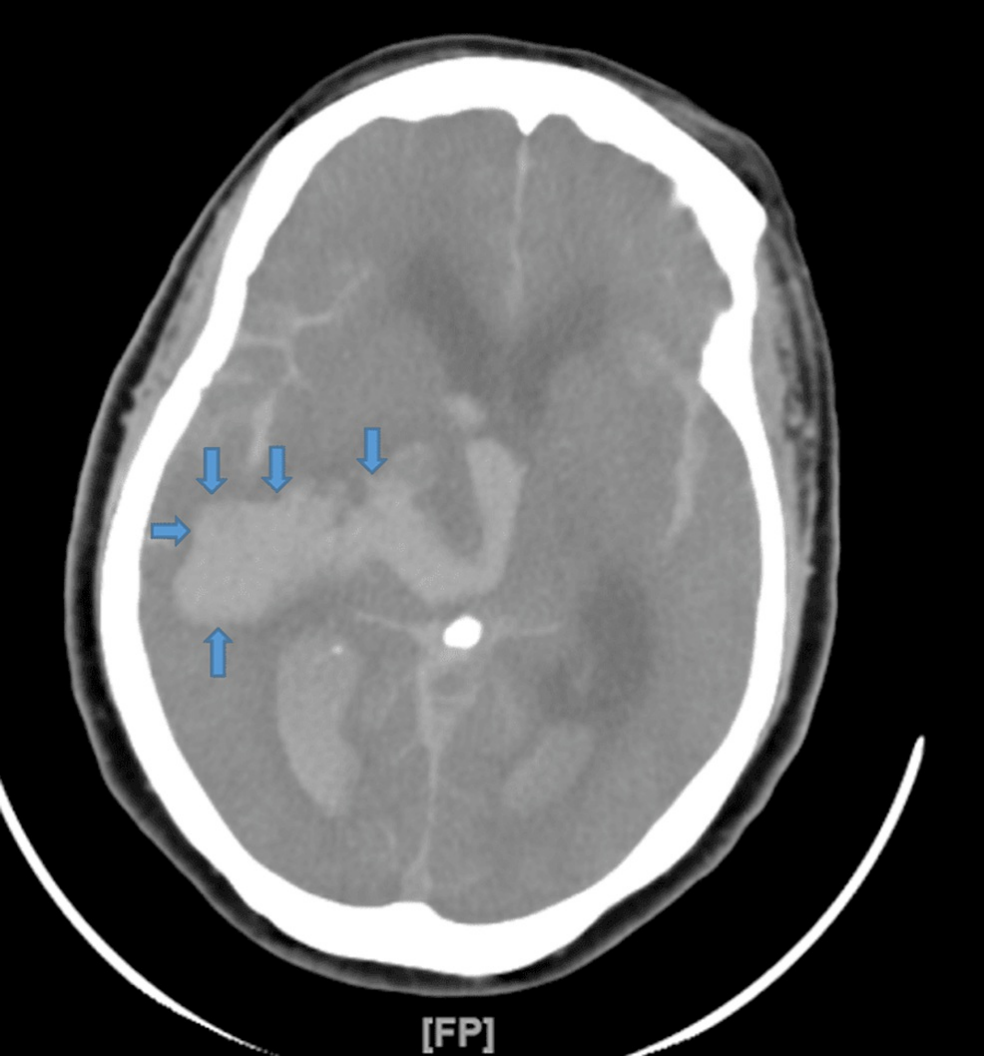
Computed tomography scan of the head without contrast revealing massive acute intracranial hemorrhage.

Our patient is an 80-year-old female with unknown past medical history who presented after loss of consciousness while shopping. The patient was unresponsive with agonal breathing on presentation and was immediately intubated and placed on mechanical ventilation. Electrocardiogram showed ST-segment elevations in leads II, III, aVF, V3, and V4 (Figure 1). Troponin I levels were 0.091 nanograms per milliliter. Emergent coronary angiogram was subsequently performed, which showed severe left ventricular apical hypokinesis with basal hypercontractility and no obstructive coronary artery disease. Computed tomography scan of the head without contrast showed massive acute ICH (Figure 2). Pupils were non-reactive, reflexes were absent, and she had no response to painful stimuli. The patient was determined to be braindead and was requiring norepinephrine and dobutamine to maintain blood pressure; the decision to perform compassionate extubation was ultimately made.

## Discussion

ICH is a life-threatening condition characterized by bleeding within the skull. TCM is a reversible cardiac disorder often triggered by emotional or physical stressors. TCM is characterized by transient left ventricular dysfunction without significant coronary artery disease. It typically presents as acute chest pain, electrocardiographic changes resembling acute coronary syndrome, and reversible left ventricular wall motion abnormalities [3-4]. The incidence of TCM following ICH is relatively rare but well-documented in the medical literature. A United States nationwide study [5] reported that the incidence rate of TCM in patients discharged with ICH between the years 2015 and 2018 was 0.27%.

The exact pathophysiology of TCM remains unclear, but catecholamine surge, microvascular dysfunction, and sympathetic overactivity have been implicated as key factors in its development [4]. Although typically associated with emotional stress, TCM can also be triggered by non-emotional stressors, including critical illnesses such as ICH [1]. ICH causes a sudden increase in intracranial pressure, leading to sympathetic activation and release of stress hormones such as catecholamines. The sudden release of catecholamines during ICH can cause myocardial stunning and dysfunction. Excessive sympathetic activation and direct catecholamine toxicity contribute to the development of TCM in this context [4]. The other proposed mechanism of TCM following ICH is neurogenic stunned myocardium; increased intracranial pressure and neurogenic stress can lead to dysregulation of autonomic pathways, resulting in abnormal cardiac function and TCM [6].

Recognizing TCM in patients with ICH is vital, as it may influence patient outcomes and treatment strategies. Differentiating TCM from other causes of cardiac dysfunction, such as myocardial infarction, is also crucial to avoid inappropriate interventions. Early electrocardiography, cardiac enzyme measurement, and coronary angiography/echocardiography should be performed in patients with ICH to assess for TCM. Serial cardiac evaluations can help monitor the recovery of ventricular function. Optimizing hemodynamic parameters, such as blood pressure and heart rate, is important in managing both ICH and TCM. Careful selection of vasoactive medications is essential to avoid exacerbating myocardial dysfunction.

## Conclusions

Here we presented a case of ICH leading to TCM. TCM is classically induced by emotional stress, but our case demonstrates that it is important for physicians to be on the lookout for TCM induced by severe illness, such as ICH, as well. A multidisciplinary team collaboration between neurologists, cardiologists, and critical care specialists is essential in managing patients with ICH-induced TCM. Comprehensive management strategies should address both the neurological and cardiac aspects of the patient's condition.
